# A multi-center, single-arm, phase II study of anlotinib plus paclitaxel and cisplatin as the first-line therapy of recurrent/advanced esophageal squamous cell carcinoma

**DOI:** 10.1186/s12916-022-02649-x

**Published:** 2022-12-08

**Authors:** Ning Li, Tao Wu, Yong-Gui Hong, Yan-Zhen Guo, Yu-Feng Cheng, Yi-Jie Ma, Liang-Yu Bie, Dong-Hai Cui, Xiao-Hui Gao, Bing-Xu Tan, Bao-Sheng Li, Su-Xia Luo, Jun-Sheng Wang

**Affiliations:** 1 Department of Medical Oncology, The Afliated Cancer Hospital of Zhengzhou University and Henan Cancer Hospital, NO. 127, Dongming Road, Jinshui District, Zhengzhou, 450008 Henan Province China; 2 Department of Internal Medicine, Anyang Tumor Hospital, No. 1 Huanbin North Road, Beiguan District, Anyang, 455000 Henan Province China; 3 Department of Medical Oncology, The First Affiliated Hospital of Henan University of Science and Technology, Luoyang, 471000 China; 4 Department of Radiotherapy, Qilu Hospital of Shandong University, Jinan, 250012 China; 5 Department of Radiotherapy, Shandong Cancer Hospital, Jinan, 250117 China

**Keywords:** Anlotinib, Paclitaxel, Cisplatin, Advanced esophageal squamous cell carcinoma, Efficacy, Safety, First-line therapy

## Abstract

**Background:**

Anlotinib, a tyrosine kinase inhibitor, has shown encouraging anti-tumor activity in esophageal squamous cell carcinoma (ESCC). This study was designed to assess the efficacy and safety of anlotinib plus paclitaxel and cisplatin (TP) as first-line therapy for advanced ESCC.

**Methods:**

In a multi-center, single-arm, phase II clinical trial, patients (aged > 18 years) with ESCC, which was judged to be locally advanced, recurrent, or metastatic, received 10 mg oral anlotinib once daily on days 1–14, 135 mg/m^2^ intravenous paclitaxel on day 1, and 60–75 mg/m^2^ intravenous cisplatin on days 1–3 every 3 weeks for a maximum of 4–6 cycles as the initial therapy in five centers in China. Subsequently, patients received anlotinib monotherapy (10 mg) as maintenance therapy until tumor progression or intolerable toxicity. The primary endpoint was progression-free survival (PFS).

**Results:**

Forty-seven patients were enrolled in this study between October 2019 and March 2021. The median follow-up was 14.04 months (IQR, 9.30–19.38). Of 46 with assessable efficacy, the median PFS and median overall survival were 8.38 months (95% CI, 6.59–10.17) and 18.53 months (95% CI, 13.11–23.95), respectively. The objective response rate was 76.1% (95% CI, 61.2–87.4%), with 4 (8.7%) complete responses and 31 (67.4%) partial responses. The disease control rate was 91.3% (95% CI, 79.2–97.6%). The median duration of response was 6.80 months (95% CI, 4.52–9.08), and 1 patient had an ongoing response for 23 months. Subgroup analysis revealed no association between clinical factors and survival or response. Of the 47 patients with assessable safety, the main grade ≥ 3 treatment-emergent adverse events (TEAEs) were neutropenia (17.0%), bone marrow suppression (12.8%), and vomiting (10.6%). No treatment-related deaths or serious TEAEs were observed. Notably, higher c-Kit levels were an independent factor for superior PFS (HR = 0.032; 95% CI, 0.002–0.606; *P* = 0.022).

**Conclusions:**

The study demonstrated a manageable safety profile and durable clinical response of anlotinib plus TP as first-line therapy in advanced ESCC, which suggested a potential therapeutic option for this population.

**Trial registration:**

ClinicalTrials.gov NCT04063683. Registered 21 August 2019.

**Supplementary Information:**

The online version contains supplementary material available at 10.1186/s12916-022-02649-x.

## Background

Esophageal cancer (EC) is the sixth most deadly cancer worldwide with almost 550,000 deaths annually [1]. Esophageal squamous cell carcinoma (ESCC), known as the predominant histological subtype of EC globally [2], commonly occurs in certain regions of Asia [3, 4], especially in China [5]. Usually, nearly half of patients with ESCC present with late-stage disease or metastasis at diagnosis [6, 7]. Platinum plus paclitaxel (TP) or fluorouracil-based combination chemotherapy, which remains the first-line therapy for unresectable recurrent, advanced, or metastatic ESCC [7, 8], provides modest benefits. However, the overall improvements are poor, short-lived, and disappointing, with a 5-year survival rate of < 20% over the last few decades [7, 9]. Therefore, there is an urgent need to develop safer and more effective therapeutic options that can break this therapeutic impasse for this population to improve their quality of life (QoL) and prolong survival.

Improved understanding of tumor immune escape and poor chemotherapy prognosis has demonstrated encouraging efficacy of immune checkpoint inhibitors (ICIs) plus chemotherapy as first-line therapy for ESCC [10–12]. As reported in the CheckMate 648 trial, a longer overall survival (OS; 13.2 months vs. 10.7 months, *P* = 0.002) was observed from nivolumab plus chemotherapy in ESCC compared to chemotherapy alone [11]. Despite the efficacy of the combined regimens being impressive, they may support modest response rates [11, 13, 14]. Furthermore, the combination may increase the incidence and severity of irAEs, which underlies significant morbidity for patients and considerable cost for healthcare systems [15]. Taken together, these regimens only benefit a certain percentage of patients and restrict their utility in clinical practice [16]. Of note, given that ESCC is known as a highly angiogenic tumor [17, 18], combination therapies with anti-angiogenic agents are valuable and attractive strategies and are worthy of further exploration [7]. The anti-tumor activity during the management of ESCC patients reported in increasing numbers of studies is far from satisfactory [17, 19]. Thus, additional investigations of combination regimens involving more promising anti-angiogenesis agents and chemotherapy remain necessary.

Anlotinib, a novel orally administered antiangiogenesis developed in China, is a small molecule tyrosine kinase inhibitor (TKI) that targets vascular endothelial growth factor receptor (VEGFR), fibroblast growth factor receptor (FGFR), platelet-derived growth factor receptor (PDGFR), and c-kit, and blocks angiogenesis [20, 21]. It has demonstrated promising anti-tumor efficacy and manageable safety profiles in a variety of solid tumors [21, 22]. Notably, a recent phase II trial demonstrated the efficacy and safety of anlotinib monotherapy as second-line therapy for previously treated advanced or metastatic ESCC patients [8]. The encouraging improvements in progression-free survival (PFS), objective response rate (ORR), and disease control rate (DCR) were observed in anlotinib over placebo [8]. Hence, anlotinib monotherapy has been recommended by the Chinese Society of Clinical Oncology (CSCO) for second-line or later treatment of metastatic ESCC at present [23]. In addition, a retrospective study revealed that anlotinib plus chemotherapy as a first- or second-line therapy achieved encouraging anti-tumor activity, satisfactory survival, and a manageable safety profile for recurrent metastatic ESCC [24], which may account for the synergistic action involving anti-angiogenesis and chemotherapy [25]. Taken together, anlotinib plus TP might be reasonably considered as a potential and encouraging first-line therapy in ESCC due to the additional multi-target anti-angiogenesis of anlotinib, while related prospective studies are lacking. Therefore, this multicenter phase II clinical trial was conducted to assess the efficacy and safety of a combination regimen as first-line therapy for ESCC patients.

## Methods

### Study design

This multi-center, single-arm, phase II clinical trial (trial registration ID: NCT04063683) was conducted at five sites in Henan and Shandong, China (Additional file 1: Table S1), and it was approved by the Ethics Review Board of each center (the Ethics Committee of Henan Cancer Hospital [No.2019315], the Ethics Committee of The First Affiliated Hospital of Henan University of Science and Technology [No. 2019-0072], the Ethics Committees of Anyang Cancer Hospital [No. AZLL022019050191030], the Ethics Committee of Shandong Cancer Hospital [No. SDZLEC2019-093-01], and the Ethics Committee of Qilu Hospital of Shandong University [No.2019134]). The primary goal of this study was to evaluate the efficacy and safety of anlotinib plus TP for patients with advanced ESCC. The study was conducted per the International Conference on Harmonization of Good Clinical Practice guidelines, the Declaration of Helsinki, and applicable local laws and regulations. The patients were informed of the investigational nature of the study and provided written informed consent before registration.

### Patient population

Eligible patients were aged between 18 and 75 years with histologically or cytologically confirmed unresectable, locally advanced, recurrent, or metastatic ESCC (TNM stage IIIb/IV; excluding mixed adenosquamous carcinoma) with at least one unresectable and measurable lesion according to Response Evaluation Criteria in Solid Tumors (RECIST) version 1.1 [26]. Patients who did not receive previous systemic therapy or who received neoadjuvant/adjuvant chemotherapy relapsed more than 6 months from the last administration of peri-operation chemotherapy or radical resection were included. Other inclusion criteria were Eastern Cooperative Oncology Group Performance Status (ECOG PS) of 0 or 1, adequate bone marrow, liver, and renal function, and a minimum of three-month predicted survival time duration.

Patients who experienced uncontrolled severe disease or had other primary malignancies at screening were ineligible for this study. Patients with complete obstruction of the esophagus, deep esophageal ulceration, prior allergy or intolerance to chemotherapeutic drugs or their excipients, or active hemorrhage involving primary lesions during the previous two months were excluded from the study. Pregnant or lactating patients, as well as patients with childbearing potential who did not use contraception if sexually active, were also excluded. Detailed exclusion criteria are listed in Additional file 2: Table S2.

### Procedures

**Fig. 1:**
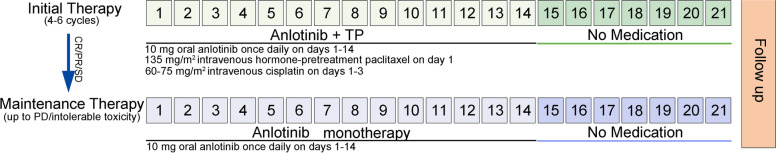
Treatment schedule (21-day cycle). The initial and maintenance therapy procedures for patients with advanced ESCC. TP, paclitaxel followed by cisplatin; CR, complete response; PR, partial response; PD, progressive disease; SD, stable disease

Eligible patients received 10 mg oral anlotinib (Anlotinib Hydrochloride Capsule AL3818, Chia Tai Tianqing Pharmaceutical Group Co., Ltd., Nanjing, China) once daily on days 1–14, 135 mg/m^2^ intravenous hormone-pretreatment paclitaxel on day 1, followed by 60–75 mg/m^2^ intravenous cisplatin on days 1–3 every 3 weeks for a maximum of 4–6 cycles as initial therapy. Patients with complete response (CR)/partial response (PR), or stable disease (SD) were then administered 10 mg oral anlotinib monotherapy once daily on days 1–14 every 21 days as maintenance therapy until progressive disease (PD), or intolerable toxicity. An overview of the therapeutic procedure was shown in Fig. 1.

Dose delay, dose reduction, or discontinuation of any drug in this combination regimen was allowed to manage toxicities. Dose reduction should prioritize anlotinib, followed by paclitaxel and cisplatin. A maximum of two dose reductions of chemotherapy were permitted when ≥ grade 3 treatment-emergent adverse events (TEAEs) occurred (paclitaxel and cisplatin: 80% or even 60% of the initial dose and then discontinuation). Notably, only one dose reduction of anlotinib (to 10 mg and then discontinuation) was allowed according to TEAEs and potential therapeutic benefits determined by investigators. If the dose was reduced or interrupted, it could not be increased in subsequent cycles. The decision to discontinue medication was based on patients’ choices and investigators’ judgments. Detailed dose titration and delayed dose criteria are listed in Additional file 3: Table S3.

### Assessments

Tumor responses were assessed by investigators using RECIST version 1.1 [26] with magnetic resonance imaging (MRI) or computed tomography (CT) at baseline and every two cycles of initial therapy or every three cycles of maintenance therapy until objective disease progression. Safety was recorded continuously for 30 days after the end of treatment and was assessed by TEAEs and severity. TEAEs were confirmed by the laboratory, hematological, and biochemical assessments, vital signs, ECOG PS performance status, physical examination, and 12-lead electrocardiogram measurements. TEAEs were graded according to the Common Terminology Criteria for Adverse Events (CTCAE), version 5.0 [27].

### Biomarker analysis

Twelve potential biomarkers (VEGF, VEGFR-1, VEGFR-2, VEGFR-3, EGFR, Ki67, CD31, FGFR-1, PDGFR-α, PDGFR-β, c-Kit, and c-Met) were analyzed using the corresponding monoclonal antibodies (Additional file 4: Table S4). The *H* score was calculated as a semiquantitative value derived from the sum of the percentages of positive cells (0–100%) multiplied by the numerical staining intensity (scale 0–3) of each, resulting in a total possible scoring range of 0 to 300 (Additional file 5: Fig. S1). The detailed methods are presented in Additional file 6: Supplementary Methods [28–32].

### Endpoints

The primary endpoint was PFS, which was defined as the time from the start of the treatment regimen to clinical progression or death from any cause, whichever came first, including initial and maintenance therapy. The secondary endpoints were OS (defined as the time between treatment initiation and death of any cause), ORR (calculated as the proportion of patients achieving CR and PR), DCR (referred to the proportion of patients with CR, PR, or SD), duration of response (DOR, defined as the duration from the day when patients firstly had CR or PR to the day they had PD firstly or death from any cause), and safety and tolerability. Safety and tolerability were assessed by TEAEs. A serious TEAE was defined as any AE that was fatal, life-threatening, required prolonged hospitalization, and resulted in persistent or significant disability/incapacity. Moreover, the exploratory endpoint was the association between potential biomarkers in tumor samples and survival differences.

### Statistical analysis

Sample size estimation was based on the primary endpoint of PFS. Approximately 25 PFS events were expected if 37 patients enrolled within a 12-month accrual period and a 12-month follow-up. This number of events would provide 80% power at a two-sided 5% significance level, assuming a true HR of 0.60 for anlotinib plus TP, corresponding to improvement in PFS from 6 months to 10.5 months based on previous trials using TP alone [33, 34]. A total of 47 patients were recruited, considering an approximate dropout incidence of 20%.

Efficacy analysis was performed in the full analysis set (FAS), which was defined as all eligible participants who received at least one dose of the study drug medication. Safety analysis was performed in the safety analysis set (SAS), which was defined as all participants who received the study drug medication at least once and had excellent safety records, regardless of eligibility. The biomarker population was defined as patients with at least one evaluable immunohistochemical biomarker.

Patient characteristics, safety outcomes, and tumor responses are summarized descriptively. Categorical variables are summarized as frequencies (percentage [%]), and continuous variables are presented as medians with interquartile range (IQR) or range. The 95% CI of the ORR and DCR were calculated using the Clopper-Pearson method. PFS, DOR, and OS were calculated using the Kaplan-Meier method with a 95% CI. Statistical comparisons of ORR according to clinical factors were performed using the chi-square test or Fisher’s exact test, while those of PFS and OS were performed using a two-sided exact log-rank test. To analyze the association of OS and PFS with corresponding biomarker expression, patients were subdivided into high or low-expression groups based on median cut-off values. To optimally balance the number of patients in the subgroups, due to the small sample size, the cutoff values for low versus high biomarker expression were the median *H* score for the study population evaluable for biomarkers. Multivariate analysis was performed using a Cox proportional-hazards model to determine survival differences and reported with an HR and 95% CI. HR < 1 implied a lower risk of progression or death in patients. Stratification of PFS or OS was performed according to biomarkers using the Kaplan-Meier method and compared using a two-sided exact log-rank test. All statistical tests were two-sided, with significance set at *P* < 0.05. All analyses were conducted using the SAS software (v.9.4; SAS Institute, Cary, NC, USA).

## Results

### Patient characteristics

**Fig. 2:**
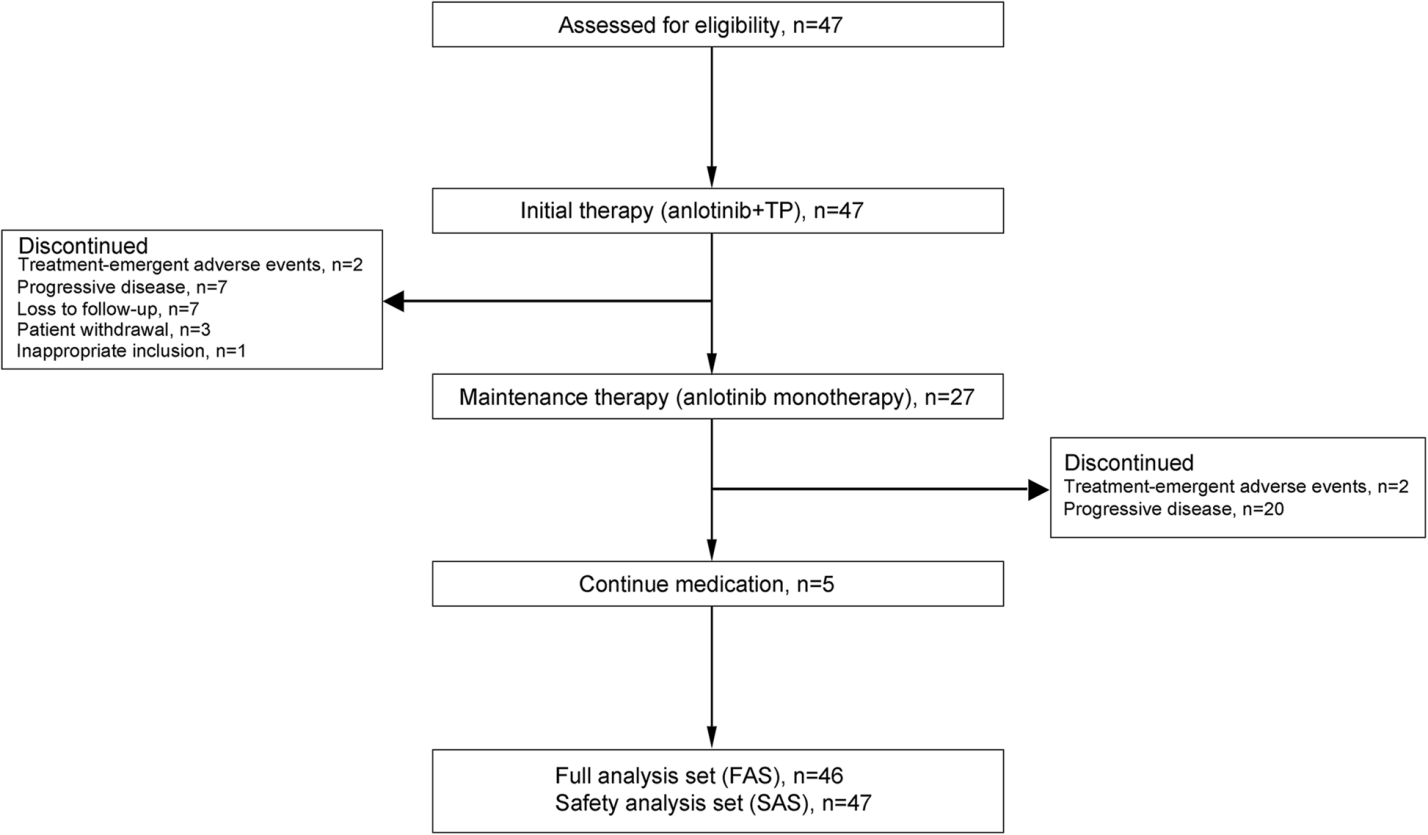
Trial profile. TP, paclitaxel followed by cisplatin

Between October 2019 and March 2021, 47 patients from 5 centers across China with a median age of 65.5 years (range, 43–75 years) with ESCC were enrolled. The median follow-up time was 14.04 months (IQR, 9.30–19.38 months). A total of 46 patients were included in the FAS, as one was excluded due to inappropriate inclusion, and all 47 patients were included in the SAS (Fig. 2). The baseline characteristics of the 46 patients are presented in Table 1. The majority of the patients were male (32/46, 69.6%). Most patients were diagnosed with TNM stage IVb (41/46, 89.1%) and had an ECOG PS performance status of 1 (31/46, 67.4%). All patients had metastasis, most commonly involving the lymph nodes (34/46, 73.9%), lungs (16/46, 34.8%), and liver (13/46, 28.3%). Nearly half of the patients (21/46, 45.7%) had their primary tumors resected.

**Table 1:** Baseline characteristics

Characteristics	All patients (*n* = 46)
Sex	
Male	32 (69.6%)
Female	14 (30.4%)
Age, years-median (range)	65.5 (43-75)
> 60	33 (71.7%)
≤ 60	13 (28.3%)
Clinical stage	
IIIb	2 (4.3%)
IVa	3 (6.5%)
IVb	41 (89.1%)
Previous surgical treatment	
Yes	21 (45.7%)
No	25 (54.3%)
Metastatic sites	
Distant lymph node	34 (73.9%)
Lung	16 (34.8%)
Liver	13 (28.3%)
Other	11 (23.9%)
Number of metastatic sites	
≤ 2	31 (67.4%)
> 2	15 (32.6%)
ECOG PS	
0	15 (32.6%)
1	31 (67.4%)

Data are presented as the median (IQR) or *n* (%). *ECOG PS* Eastern Cooperative Oncology Group Performance Score

### Treatment

Twenty-seven (57.4%) patients who achieved CR/PR/SD following initial therapy subsequently received maintenance therapy. At the data cut-off (23 January 2022), 5 (10.6%) patients were still on first-line therapy, while 42 (89.4%) patients discontinued medication due to disease progression (57.4%), loss to follow-up (14.9%), intolerable TEAEs (8.5%), withdrawal (6.4%), and inappropriate inclusion (2.1%). Eleven (23.9%) patients initiated second-line therapy, including three (6.5%) immunochemotherapy, one (2.2%) immunotherapy, three (6.5%) chemotherapy, one (2.2%) concurrent chemoradiation, and three (6.5%) surgery. Twenty-one (45.7%) patients did not receive follow-up treatment, and nine (19.6%) had an unknown status.

### Efficacy

**Fig. 3:**
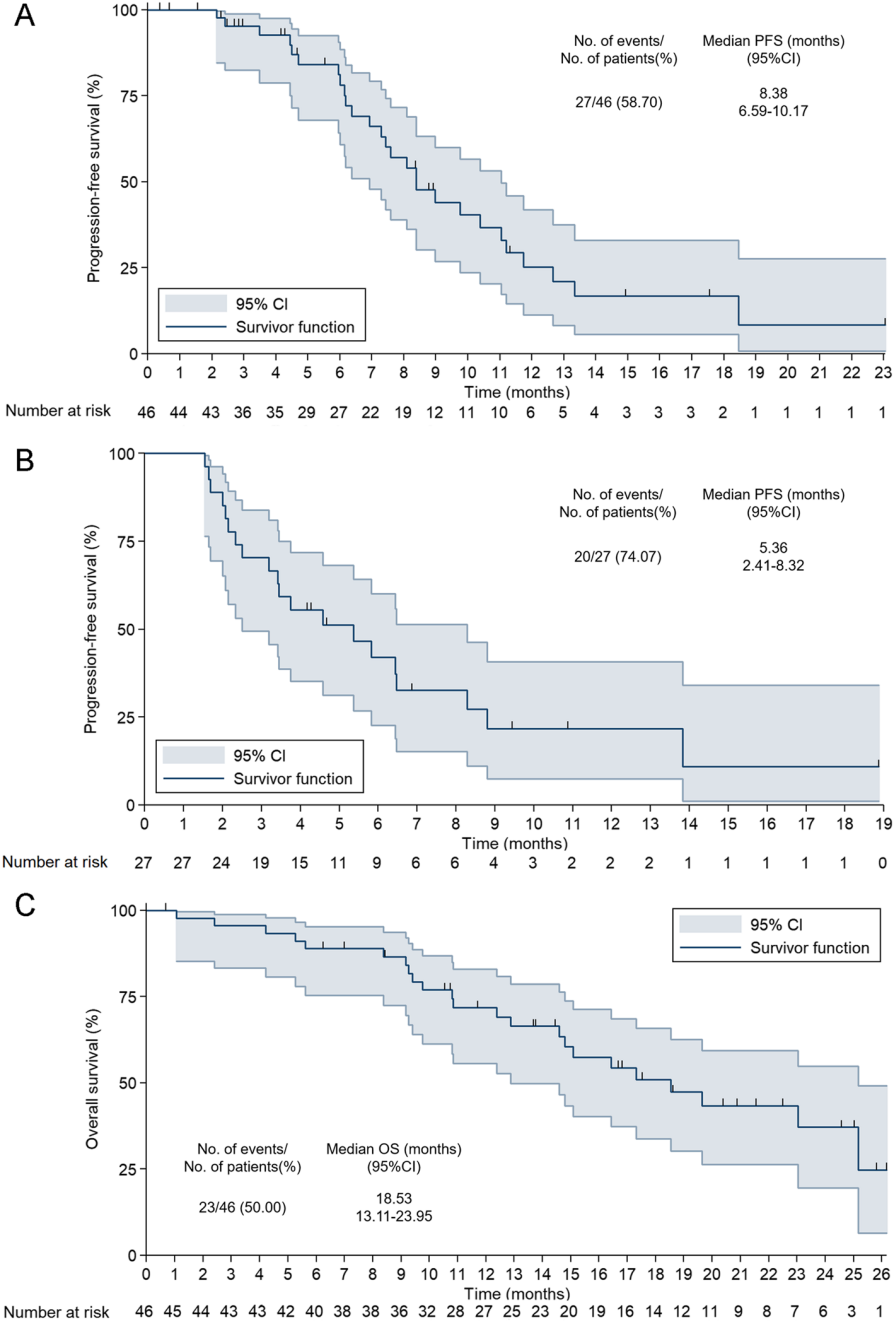
Kaplan-Meier analyses of survival in the initial and maintenance therapy. **A** Progression-free survival of initial therapy (full analysis set, *n* = 46). **B** Progression-free survival of maintenance therapy (*n* = 27). **C** Overall survival of initial therapy (full analysis set, *n* = 46). CI, confidence interval

PFS (disease progression or death) was observed in 27 patients (58.7 %). The median PFS (mPFS) was 8.38 months (95% CI, 6.59–10.17, Fig. 3A), with 6- and 12-month PFS rates of 81.12% (95% CI, 64.26–90.58%) and 25.17% (95% CI, 11.26–41.85%), respectively. The PFS of maintenance therapy was also analyzed, which was named PFS2 and was defined as the time from maintenance therapy to disease progression or death from any cause. The median PFS2 was 5.36 months (95% CI, 2.41–8.32), and the 6- and 12-month PFS rates were 41.96% (95% CI, 22.69–60.14%) and 21.76% (95% CI, 7.46–40.82%), respectively (Fig. 3B). Furthermore, OS analysis was also performed due to a sufficiently long follow-up period. Twenty-three OS events (50.0%) occurred in the FAS population. The median OS (mOS) was 18.53 months (95% CI, 13.11–23.95), with 12- and 24-month OS rates of 71.80% (95% CI, 55.61–82.95%) and 37.21% (95% CI, 19.61–54.88%), respectively (Fig. 3C).

**Fig. 4:**
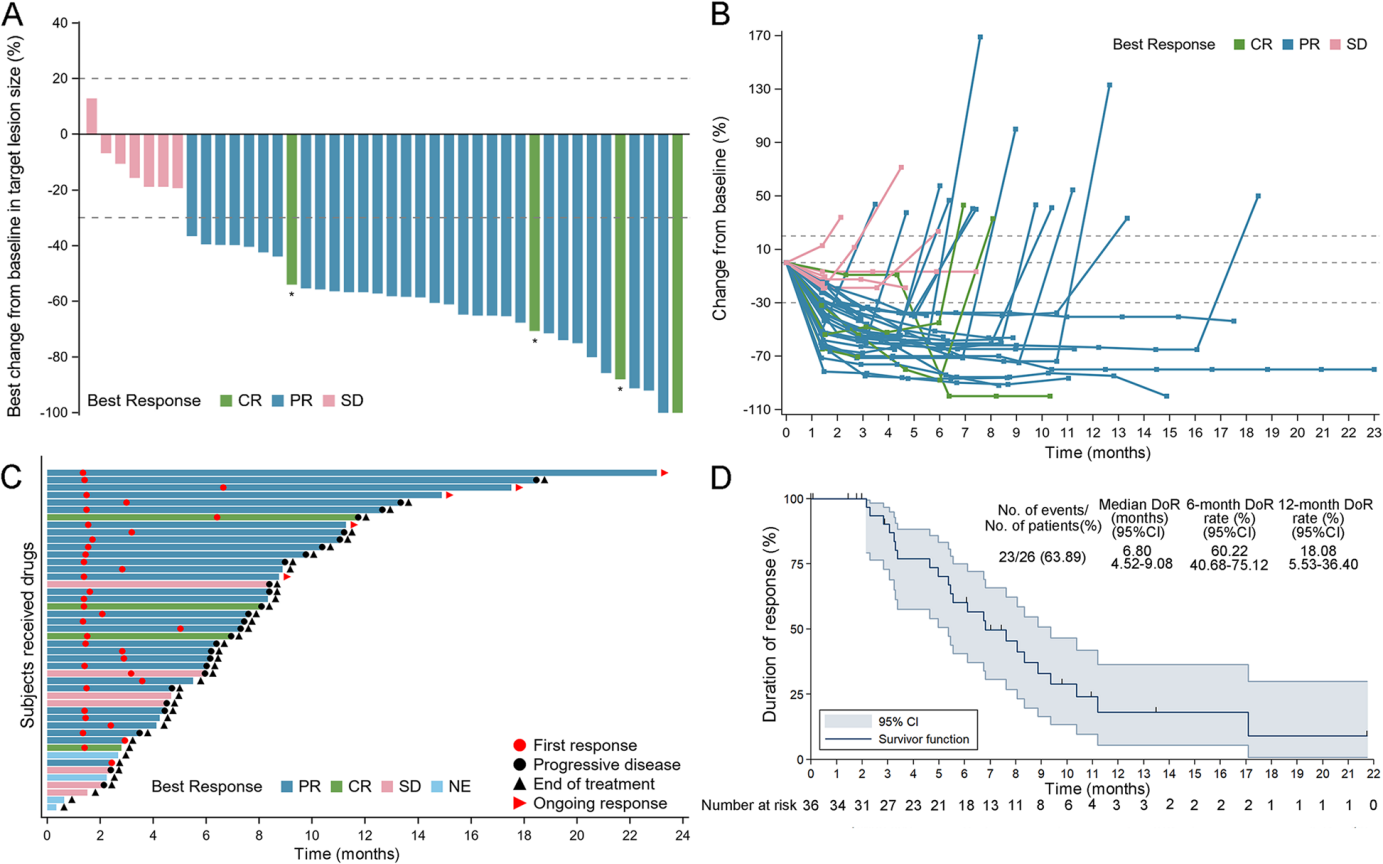
Tumor response. **A** Waterfall plot of tumor size change from baseline to maximum percentage in each patient as per RECIST version 1.1. **B** Longitudinal change in tumor size from baseline. **C** Time to response and duration of response. **D** Duration of response for patients with advanced ESCC. CR, complete response; PR, partial response; PD, progressive disease; SD, stable disease; NE, not evaluable; ESCC, esophageal squamous cell carcinoma. Asterisk symbol (*) indicates the following: CR was confirmed with the disappearance of all target lesions and any pathological lymph nodes (whether target or non-target) must have a reduction in short axis to < 10 mm

In the FAS population, ORR was observed in 35 patients (76.1%; 95% CI, 61.2–87.4%), including four (8.7%, Additional file 7: Table S5) CR and 31 (67.4%) PR, per RECIST v.1.1, respectively (Table 2). The number of patients who achieved SD was 7 (15.2%); thus, the DCR was 91.3% (95% CI, 79.2–97.6%). Of 42 patients with evaluable imaging, 41 (89.1%) achieved tumor shrinkage and decreased target lesion size from baseline (Fig. 4A, B). The treatment duration for all 46 participants is shown in Fig. 4C. Of the 36 (78.3%) patients with responses, the median DOR was 6.8 months (95% CI, 4.52–9.08, Fig. 4D). The first response was achieved within 2 months, and 1 patient had an ongoing response for 23 months.

**Table 2:** Anti-tumor activity of anlotinib plus TP regime in the first-line therapy for ESCC

Best responses	All patients (*n* = 46)
CR	4 (8.7%)
PR	31 (67.4%)
SD	7 (15.2%)
PD NE	0 4 (8.7%)
ORR	35 (76.1%, 61.2–87.4%)
DCR	42 (91.3%, 79.2–97.6%)

Data are presented as *n* (%) or *n* (%, 95% confidence interval)

*CR* complete response, *PR* partial response, *SD* stable disease, *PD* progressive disease, *NE* not evaluable, *ORR* overall response rate, *DCR* disease control rate

### Subgroup analysis

Clinical factors including age, sex, ECOG PS, primary tumor resected, metastatic sites, numbers of metastatic sites, and distant metastasis were not significantly associated with PFS (Additional file 8: Table S6), OS (Additional file 9: Table S7), and ORR (Additional file 10: Table S8). Of note, the mOS tended to be longer in patients without liver metastasis than in those with liver metastasis (23.03 months vs.14.78 months, P = 0.041), while further analysis was not performed due to the immature data.

### Biomarker analysis

**Fig. 5:**
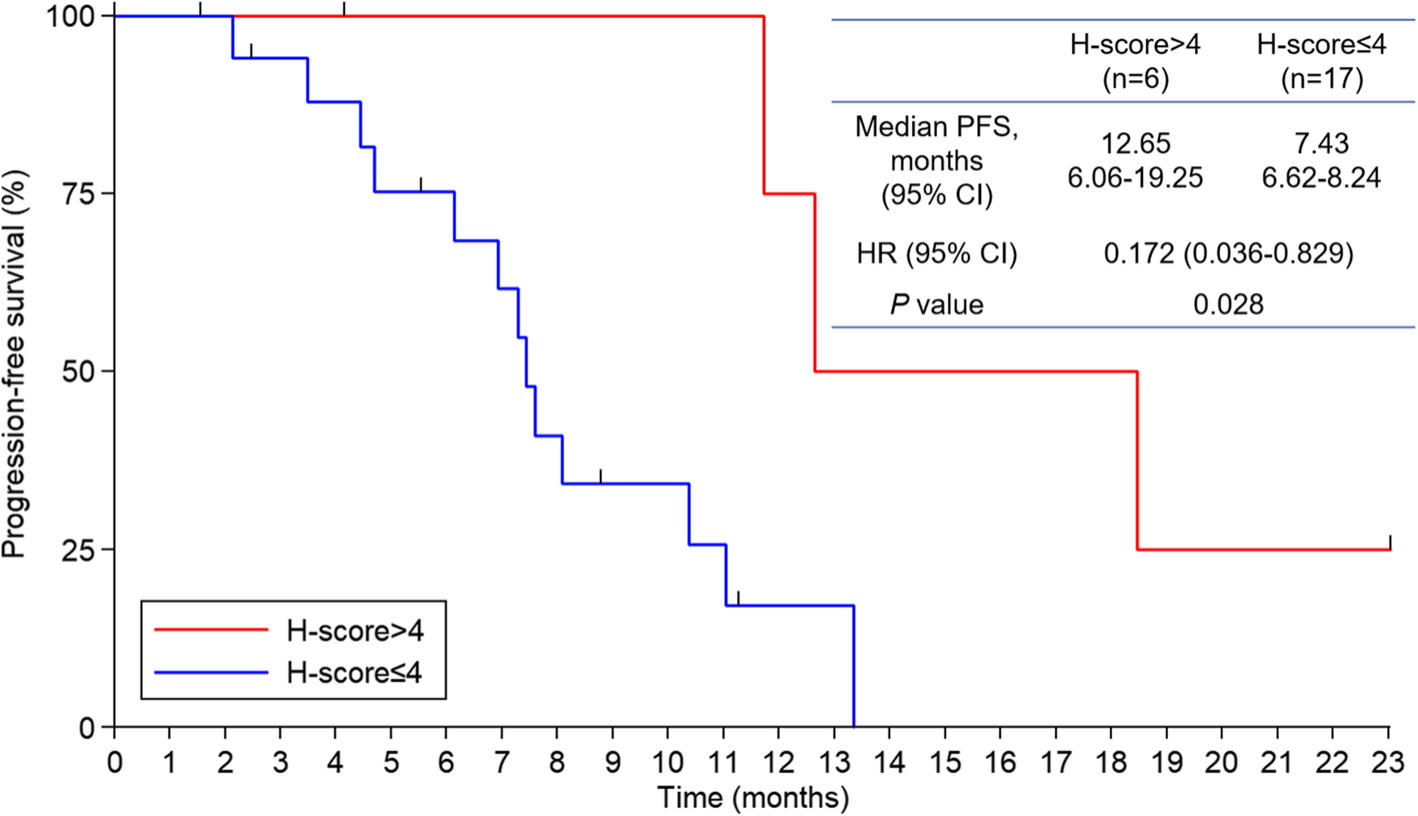
Progression-free survival (PFS, c-Kit population). PFS was stratified by c-Kit levels (*H*-score ≤ 4 vs. *H*-score > 4). CI, confidence interval; HR, hazard ratio

The biomarker population was comprised of 23 patients. Taking c-Kit as an example, all 23 patients had evaluable data for c-Kit (c-Kit population), 17 patients had low c-Kit expression (*H* score ≤ 4), and 6 patients had high c-Kit expression (*H* score > 4). Multivariate analysis demonstrated that there was a trend for greater efficacy in the high c-Kit expression group than in the low c-Kit expression group, as reflected by a smaller HR estimate (HR = 0.032; 95% CI, 0.002–0.606; *P* = 0.022). Additionally, further subgroup analysis verified that patients in the high c-Kit expression group had a longer mPFS than those in the low c-Kit expression group (12.65 months vs. 7.43 months; *P* = 0.028, Fig. 5). However, no associations between other biomarkers and PFS (Additional file 11: Table S9) or OS (Additional file 12: Table S10) were observed.

### Safety and tolerability

The majority (46/47, 97.9%) of the safety population experienced at least one TEAE. Common TEAEs included nausea (78.7%), vomiting (66.0%), decreased hemoglobin levels (51.1%), acid reflux (51.1%), leukopenia (40.4%), swallowing difficulty (38.3%), thrombocytopenia (38.3%), hypokalemia (29.8%), neutropenia (27.7%), bone marrow suppression (25.5%), and increased alanine aminotransferase (21.3%). The most frequent TEAEs (> 10%) and grade ≥ 3 TEAEs (> 3%) are presented in Table 3. Grade 3 or higher TEAEs occurred in 23 (48.9%) patients and mainly included neutropenia (17.0%), bone marrow suppression (12.8%), nausea (10.6%), and vomiting (10.6%). Dose reduction of anlotinib occurred in four (8.5%) patients due to TEAEs, and two (4.3%) patients required chemotherapeutic dose reduction. Three patients (6.4%) discontinued treatment due to TEAEs during initial therapy. Furthermore, two (4.3%) patients permanently discontinued the combination regimen due to grade 4 thrombocytopenia and grade 3 upper gastrointestinal hemorrhage. No grade 5 TEAEs, serious TEAEs, or TEAE-related deaths were observed.

**Table 3:** Treatment-emergent adverse events (TEAEs) occurring in ≥ 10% of patients and ≥ grade 3 TEAEs in > 3%

Safety population (*n* = 47)		
	Any grade	Grade 3 or more
Nausea	37 (78.7%)	5 (10.6%)
Vomiting	31 (66.0%)	5 (10.6%)
Decreased hemoglobin	24 (51.1%)	-
Acid reflux	24 (51.1%)	3 (6.4%)
Leukopenia	19 (40.4%)	3 (6.4%)
Swallowing difficulty	18 (38.3%)	3 (6.4%)
Thrombocytopenia	18 (38.3%)	2 (4.3%)
Hypokalemia	14 (29.8%)	3 (6.4%)
Neutropenia	13 (27.7%)	8 (17.0%)
Bone marrow suppression	12 (25.5%)	6 (12.8%)
Increased alanine aminotransferase	10 (21.3%)	-
Fatigue	10 (21.3%)	-
Proteinuria	9 (19.1%)	-
Lymphocytopenia	7 (14.9%)	3 (6.4%)
Constipation	7 (14.9%)	-
Hyponatremia	7 (14.9%)	-
Anorexia	7 (14.9%)	-
Diarrhea	6 (12.8%)	2 (4.3%)
Hypochloridemia	6 (12.8%)	-
Hypertension	6 (12.8%)	-
Hypocalcemia	5 (10.6%)	-
Increased indirect bilirubin	5 (10.6%)	-
Lower limb ache	5 (10.6%)	-
Increased aspartate aminotransferase	5 (10.6%)	-
Chest pain	4 (8.5%)	3 (6.4%)

Data are *n* (%)

During maintenance therapy, no patients experienced TEAEs leading to dose reduction, interruptions, or delays. Only 2 (4.3%) patients experienced permanent discontinuation, including acute kidney injury (grade 3) and diarrhea (grade 4). No fatal TEAEs occurred during maintenance therapy.

## Discussion

The management of patients with ESCC remains challenging given the aggressiveness of the disease and the limited choice of effective anti-tumor agents [35]. To the best of our knowledge, this was the first study to confirm the feasibility of manageable toxicities of anlotinib plus TP as first-line therapy for advanced ESCC, with an mPFS of 8.38 months.

With respect to efficacy, the mPFS reported in previous studies evaluating TP alone as the upfront treatment for advanced ESCC was 5.6 months, with an ORR of 56.5% [36]. In contrast, a longer mPFS of 8.38 months was achieved, and 76.1% of the patients achieved an objective response under this combination regimen. As the regimens of these trials were similar to the chemotherapy backbone in the present study, the notable contrast in efficacy, reflecting remarkable tumor regression in the current trial, strongly supports the hypothesis of synergy between anti-angiogenesis and chemotherapy. In addition, the improvement in mPFS was encouraging compared to the results from previous studies using ICIs (that is, pembrolizumab, nivolizumab, camrelizumab, and sintilimab) plus chemotherapy (5.8–7.2 months) as first-line therapy [14, 37–40], and the mOS of 18.53 months in our study was also promising. These inspiring outcomes suggest that early application of this combination regimen could prolong the survival of patients with advanced ESCC. Interestingly, although the improvement in mDOR (6.80 months) observed was modest compared with previous studies [37–39], a high response rate (76.1%) was achieved in the present study. It was reasonably assumed that anlotinib may inhibit angiogenesis more comprehensively owing to its characteristics of multiple targets, as well as its ability to sensitize paclitaxel and reduce chemotherapy resistance [41, 42]. All patients enrolled in the present study were in the advanced stage (IIIb, IVa, and IVb) and had metastases. However, 91.3% of patients still had disease control, and four patients attained CR. The overall response and survival using this combination seem favorable, especially when considered for all enrolled patients with advanced-stage tumors. In addition, the impressive response and survival may demonstrate the relatively broad applicability of the proposed regimen owing to the poor and significantly different baseline status, such as the fact that nearly half of the patients had primary tumors resected. PFS, OS, and ORR were not associated with the baseline characteristics of patients in the subgroup analysis. Although OS may be related to liver metastasis, further analysis was not performed because of immature data. Taken together, these results support the feasibility of using anlotinib plus TP as first-line therapy for various ESCC populations. Notably, the JUPITER-06 trial demonstrated that toripalimab alone following initial therapy was a novel and intriguing chance to improve survival due to the lack of evidence of standard maintenance therapy in ESCC [43]. Nevertheless, the popularity was challenging owing to the disadvantages of remaining irAEs, modest efficacy, and injection therapy. Interestingly, one patient achieved an ongoing response for 23 months, and the mPFS2 was 5.36 months in our study, which suggested additional benefits of oral anlotinib combined with TP followed by anlotinib maintenance for advanced ESCC. However, further randomized controlled trials of whether anlotinib monotherapy could be applied as a maintenance regimen alone are required because of the lack of evidence regarding maintenance therapy for PD patients. In addition, the potential impact of anlotinib plus TP as initial therapy followed by anlotinib alone as maintenance therapy for subsequent line therapy was investigated. At the data cut-off, 5 patients were still on first-line therapy, and 11 received various second-line therapies, including immunochemotherapy, immunotherapy, chemotherapy, concurrent chemoradiation, and surgery. It further suggested that this proposed first-line therapy had promising antitumor activity and might support potential benefits for numerous options of later treatment.

The identification of predictive biomarkers is particularly important for the selection of optimal candidates for targeted therapy, which also applies to anlotinib [22]. Thus, we conducted a comprehensive analysis of the associations between potentially sensitive biomarkers and the treatment effects of the proposed regimen due to the absence of relevant ESCC studies. The results showed that the c-Kit level was an independent factor for better PFS in patients with advanced ESCC. Subgroup analysis verified that the mPFS of patients with low and high c-Kit expression was 7.43 and 12.65 months, respectively. Although previous studies have shown a correlation between clinical efficacy outcomes and c-Kit levels in patients with gastrointestinal tumors [44], this conclusion should be interpreted with caution because of the relatively small sample size in the present study and the complexity of tumor-microenvironment interactions. However, this trial could not differentiate whether the relationship was prognostic or predictive due to its single-arm design. In contrast, no associations between other biomarkers and PFS and OS were observed in our study, which may be owing to the small sample size. Importantly, all potential biomarkers, including c-Kit, for monitoring treatment responses to this regimen in patients with advanced ESCC, require additional observation and confirmation in randomized prospective trials with a larger sample size, which may allow further customization of treatments and prediction of individualized therapeutic responses.

It is critical to consider TEAEs when patients receive potentially effective combination regimens [42]. Common TEAEs observed in our study, such as nausea, vomiting, and bone marrow suppression, were usually tolerable and manageable and disappeared rapidly after symptomatic treatment. The reported non-hematological TEAEs were known, uncommon, and similar to those of the TP regimen [34], indicating that the majority of these TEAEs mostly resulted from the TP regimen alone, and anlotinib might not increase the risk of non-hematological toxicity. Importantly, only five patients experienced ≥ grade 3 nausea, while none exhibited anorexia or asthenia. The non-hematological toxicities under this regimen were generally mild, controllable, and tolerable, and resolved soon after symptomatic treatment. Besides, only two patients discontinued treatment due to grade 4 thrombocytopenia and grade 3 upper gastrointestinal hemorrhage during initial therapy. Overall, the results suggested manageable tolerability of anlotinib plus TP for advanced ESCC. In addition, the primary TEAEs of anlotinib identified in the previous review [22], including hypertension and proteinuria, were observed in our study, which was mild, tolerable, and manageable (grades 1–2). Furthermore, no new safety signal [8], and dose reduction were observed during maintenance therapy, suggesting that anlotinib alone might be a novel, suitable, and potential maintenance therapy regimen for testing. Only two patients experienced permanent discontinuation due to TEAEs, while TEAEs disappeared quickly after symptomatic treatment, and no treatment-related deaths occurred.

Certain limitations of our study should be acknowledged, as is typical of early-phase trials. First, this was a single-arm, non-randomized, phase II study with a relatively limited sample size, lacking comparison with other existing regimens. Second, the results of maintenance therapy may be biased by imperfect design. Owing to the relatively immature data surrounding maintenance therapy, further evaluation is needed. Third, this non-global study with data from multiple centers was conducted only in China, which might affect the generalizability of the results to broader populations. Nevertheless, the present study confirmed that anlotinib plus TP could be considered a promising first-line therapy for ESCC. Further evaluation of this combination therapy in a randomized phase III trial with a larger sample size and longer follow-up period is warranted in the near future. The combination of chemotherapy and immunotherapy is changing the paradigm in the field of advanced ESCC [13]. Interestingly, anti-angiogenesis, immunotherapy, and chemotherapy have shown encouraging efficacy in patients with ESCC based on recent studies [35]. Of note, a recent case report demonstrated that anlotinib plus chemotherapy as an effective fourth-line therapy provided novel perspectives when postoperative ESCC relapsed following immunotherapy failure due to resistance [45]. Accordingly, anlotinib has the potential to be a partner for the combination of chemotherapy and immunotherapy, improving the survival of patients with ESCC, and further clinical trials (NCT05013697) are being conducted. Notably, since a relevant proportion of patients did not benefit from ICIs, biomarker-driven selection of immunotherapy responders and non-responders would minimize unnecessary exposure of patients to potentially permanent and life-threatening immune-related toxicities and optimize treatment personalization [46]. Therefore, our future studies may benefit from a validated biomarker (programmed death-ligand 1 [PD-L1]) assessed by combined positive score (CPS) and tumor proportion score (TPS), which will be helpful for direction of better achievement of optimal selection of patients and individualized treatment.

## Conclusions

Anlotinib plus TP showed encouraging anti-tumor activity and manageable safety profiles in the first-line therapy of patients with unresectable locally advanced or recurrent/metastatic ESCC, providing a feasible and well-tolerated treatment option for this population. In addition, the findings could be considered as pilot evidence for additional insight into the application of anlotinib as a maintenance medication in patients with ESCC who benefited from initial therapy. Further evaluation of this new combination regimen in a larger randomized clinical trial is warranted in the near future.

## Supplementary Information

**Additional file 1:** **Table S1.** All centers participating in the study

**Additional file 2:** **Table S2.** Detailed exclusion criteria

**Additional file 3:** **Table S3.** Detailed dose titration and delayed dose criteria of anlotinib

**Additional file 4:** **Table S4.** The information of primary antibodies

**Additional file 5:** **Figure S1.** Representative immunohistochemical staining of 12 potential biomarkers

**Additional file 6.** Supplementary Methods for immunohistochemical staining and scoring of potential biomarkers

**Additional file 7: Table S5.** Patients who achieved complete response (*n* = 4)

**Additional file 8:** **Table S6.** Subgroup analysis of the relation between clinical factors and progression-free survival (PFS)

**Additional file 9:** **Table S7.** Subgroup analysis of the relation between clinical factors and overall survival (OS)

**Additional file 10:** **Table S8.** Subgroup analysis of the correlation between clinical factors and overall response rate (ORR)

**Additional file 11:** **Table S9.** Multivariate analysis of the correlation between biomarkers and progression-free survival (PFS)

**Additional file 12:** **Table S10.** Multivariate analysis of the correlation between biomarkers and overall survival (OS)

## Data Availability

The datasets used and/or analyzed during the current study are available from the corresponding author on reasonable request.

## Abbreviations

ESCC: Esophageal squamous cell carcinoma
TP: Paclitaxel and cisplatin
PFS: Progression-free survival
TEAEs: Treatment-emergent adverse events
EC: Esophageal cancer
QoL: Quality of life
ICIs: Immune checkpoint inhibitors
TKI: Tyrosine kinase inhibitor
VEGFR: Vascular endothelial growth factor receptor
FGFR: Fibroblast growth factor receptor
PDGFR: Platelet-derived growth factor receptors
ORR: Objective response rate
DCR: Disease control rate
CSCO: Chinese Society of Clinical Oncology
RECIST: Response Evaluation Criteria in Solid Tumors
ECOG PS: Eastern Cooperative Oncology Group Performance Status
CR: Complete response
PR: Partial response
SD: Stable disease
PD: Progressive disease
MRI: Magnetic resonance imaging
CT: Computed tomography
CTCAE: Common Terminology Criteria for Adverse Events
OS: Overall survival
DOR: Duration of response
HR: Hazard ratio
FAS: Full analysis set
SAS: Safety analysis set
IQR: Interquartile range
CI: Confidence interval
mPFS: Median PFS
PD-L1: Programmed death-ligand 1
CPS: Combined positive score
TPS: Tumor proportion score
