# Supplementary material for: A multi-center, single-arm, phase II study of anlotinib plus paclitaxel and cisplatin as the first-line therapy of recurrent/advanced esophageal squamous cell carcinoma
Source: BMC Med. 2022 Dec 8;20:472. doi: 10.1186/s12916-022-02649-x (PMC9733004; doi:10.1186/s12916-022-02649-x)
Supplement: Supplementary file 1 — Additional file 1: Table S1. All centers participating in the study [file 12916_2022_2649_MOESM1_ESM.docx]

**Table S1.** **All centers participating in the study**

| Name of centers | Investigator | No. of patients |
| --- | --- | --- |
| Henan Cancer Hospital | Su-Xia, Luo | 9 |
| The First Affiliated Hospital of Henan University of Science and Technology | Yan-Zhen, Guo | 5 |
| Anyang Cancer Hospital | Jun-Sheng, Wang | 32 |
| Shandong Cancer Hospital | Bao-Sheng, Li | 0 |
| Qilu Hospital of Shandong University | Yu-Feng, Cheng | 1 |
| Total |  | 47 |

Data are n.
