# Supplementary material for: A multi-center, single-arm, phase II study of anlotinib plus paclitaxel and cisplatin as the first-line therapy of recurrent/advanced esophageal squamous cell carcinoma
Source: BMC Med. 2022 Dec 8;20:472. doi: 10.1186/s12916-022-02649-x (PMC9733004; doi:10.1186/s12916-022-02649-x)
Supplement: Supplementary file 2 — Additional file 2: Table S2. Detailed exclusion criteria [file 12916_2022_2649_MOESM2_ESM.docx]

**Table S2. Detailed exclusion criteria**

| Exclusion criteria |
| --- |
| Complete obstruction of the esophagus |
| Deep esophageal ulceration with an increased risk of bleeding |
| Unreduced esophageal lesions following radiotherapy without surgery |
| Previous esophageal or tracheal stent placement |
| High risk of bleeding or perforation due to the tumor’s obvious invasion of the adjacent organs or the presence of a formed fistula |
| Prior allergy or intolerance to both chemotherapeutic drugs or their excipients |
| The burden of liver metastases accounting for > 50% of the total liver |
| Recurrence/metastasis using adjuvant chemotherapy with paclitaxel within 1 year |
| Non-healing wounds or fractures for a long time |
| Factors affecting oral medication (swallowing difficulty, chronic diarrhea, intestinal obstruction, et al.) |
| Interstitial lung disease (ILD) with steroid hormone therapy |
| Active tuberculosis |
| Significant malnutrition |
| Immune deficiency |
| HIV-positive |
| Other acquired and congenital immunodeficiency diseases |
| Previous organ transplantation |
| Symptomatic CNS metastases and/or carcinomatous meningitis |
| Respiratory dysfunction due to pleural effusion |
| Major surgical operation within 4 weeks prior to the study or during study treatment |
| Ascites requiring treatment |
| Uncontrolled metabolic disorders or other non-malignant organ or systemic diseases or secondary reactions to cancer |
| A history of psychotropic substance abuse with the inability to quit, or dysphrenia |
| Pregnant or lactating, childbearing potential and not using contraception if sexually active |
| Gastrointestinal perforation and/or fistula, or arterial and venous thrombosis within 6 months prior to the study |
| Other primary malignancies |
| Active hemorrhage at the primary lesions during the previous 2 months, > grade 1 pulmonary hemorrhage or > grade 2 other bleeding during the previous 4 weeks, evidence of bleeding diathesis or coagulopathy or bleeding tendency |
| Severe concomitant disease in the opinion of investigators |
| Active or uncontrolled severe infection |
| Liver diseases (cirrhosis, decompensated liver disease, chronic active hepatitis) |
| Any severe and/or uncontrolled disease |
| Poor diabetes control (fasting blood glucose [FBG] > 10 mmol/L) |
| Urinary protein ≥ ++, and confirmed 24-hour urinary protein > 1.0 g |
| Poor blood pressure control (systolic blood pressure ≥ 150 mmHg or diastolic blood pressure ≥ 100 mmHg) |
| Myocardial ischemia or myocardial infarction, arrhythmia with > class II, left ventricular ejection fraction (LVEF) < 50%, cardiac insufficiency in New York Heart Association (NYHA) functional class III-IV |
