# Supplementary material for: A multi-center, single-arm, phase II study of anlotinib plus paclitaxel and cisplatin as the first-line therapy of recurrent/advanced esophageal squamous cell carcinoma
Source: BMC Med. 2022 Dec 8;20:472. doi: 10.1186/s12916-022-02649-x (PMC9733004; doi:10.1186/s12916-022-02649-x)
Supplement: Supplementary file 3 — Additional file 3: Table S3. Detailed dose titration and delayed dose criteria of anlotinib [file 12916_2022_2649_MOESM3_ESM.docx]

**Table S3. Detailed dose titration and delayed dose criteria of anlotinib**

| Treatment-emergent adverse events | Administration time | Dose titration |
| --- | --- | --- |
| Grade 0-2 | On-time delivery/Delay delivery | Normal dose |
| Grade 3 | Delay delivery | Reduce dose if TEAEs < grade 2 |
| Grade 4 | Delay delivery | Reduce dose or discontinuation per investigators if TEAEs < grade 2 |
| Abnormal liver function (increased ALT, AST, or total bilirubin)  Grade 1  Grade 2 (normal baseline)  Grade 2 (abnormal baseline)  Grade 3  Grade 4  Proteinuria  Grade 1 (urine protein < 1.0 g [24 h])  Grade 2 (1.0 g ≤ urine protein < 2.0 g [24 h])  Grade 2 (2.0 g ≤ urine protein < 3.5 g [24 h])  Grade 3 (urine protein ≥ 3.5 g [24 h])  Decreased platelet count  Grade 1 (75×10^9^/L ≤ platelet count < 100×10^9^/L)  Grade 2 (50×10^9^/L ≤ platelet count < 75×10^9^/L)  Grade 3 (25×10^9^/L ≤ platelet count < 50×10^9^/L)  Grade 4 (platelet count < 25×10^9^/L)  Bleeding  Grade 1  Grade 2  ≥ Grade 3 | On-time delivery  Delay delivery  On-time delivery  Delay delivery  Discontinuation  On-time delivery  On-time delivery  Delay delivery till TEAEs < grade 2 within 2 weeks  Delay delivery till TEAEs < grade 2 within 2 weeks  Delay delivery  Delay delivery  Delay delivery  Discontinuation  On-time delivery  Delay delivery  Discontinuation | Normal dose  Reduce dose if TEAEs < grade 2  Normal dose  Reduce dose if TEAEs < grade 2  Discontinuation  Normal dose  Normal dose  Reduce dose or discontinuation if the third occurs  Reduce dose or discontinuation if the third occurs  Normal dose if platelet count returned to baseline or normal values  Normal dose if platelet count returned to baseline or normal values within 1 week, otherwise reduce the dose  Reduce dose if platelet count returned to baseline or normal values within 2 weeks  Discontinuation  Normal dose  Reduce dose if TEAEs < grade 2 within 2 weeks  Discontinuation |
