# Supplementary material for: A multi-center, single-arm, phase II study of anlotinib plus paclitaxel and cisplatin as the first-line therapy of recurrent/advanced esophageal squamous cell carcinoma
Source: BMC Med. 2022 Dec 8;20:472. doi: 10.1186/s12916-022-02649-x (PMC9733004; doi:10.1186/s12916-022-02649-x)
Supplement: Supplementary file 4 — Additional file 4: Table S4. The information of primary antibodies [file 12916_2022_2649_MOESM4_ESM.docx]

**Table S4. The information of primary antibodies**

| Primary antibody | Species | Source | Dilution |
| --- | --- | --- | --- |
| VEGFR-1/FLT-1 polyclonal antibody | Rabbit | Proteintech Group | 1:50 |
| VEGF receptor 2 polyclonal antibody | Rabbit | Proteintech Group | 1:50 |
| VEGF receptor 3 polyclonal antibody | Rabbit | Abcam | 1:50 |
| VEGF polyclonal antibody | Rabbit | Proteintech Group | 1:50 |
| EGFR monoclonal antibody | Mouse | Proteintech Group | 1:1000 |
| FGFR1 monoclonal antibody | Mouse | Proteintech Group | 1:250 |
| Ki67 monoclonal [SP6] antibody | Rabbit | Abcam | 1:200 |
| PDGFR-α monoclonal [EPR22059-270] antibody | Rabbit | Abcam | 1:250 |
| PDGFR-β monoclonal [42G12] antibody | Mouse | Abcam | 1:10 |
| CD31 polyclonal antibody | Rabbit | Proteintech Group | 1:800 |
| c-Kit/CD117 polyclonal antibody | Rabbit | Proteintech Group | 1:250 |
| c-Met (cytoplasmic) polyclonal antibody | Rabbit | Proteintech Group | 1:200 |

VEGFR = vascular endothelial growth factor receptor; FLT = fms-like tyrosine kinase; VEGF = vascular endothelial growth factor; EGFR = epidermal growth factor receptor; FGFR = fibroblast growth factor receptor; PDGFR = platelet-derived growth factor receptor.
