# Supplementary figures and images for: A multi-center, single-arm, phase II study of anlotinib plus paclitaxel and cisplatin as the first-line therapy of recurrent/advanced esophageal squamous cell carcinoma
Source: BMC Med. 2022 Dec 8;20:472. doi: 10.1186/s12916-022-02649-x (PMC9733004; doi:10.1186/s12916-022-02649-x)

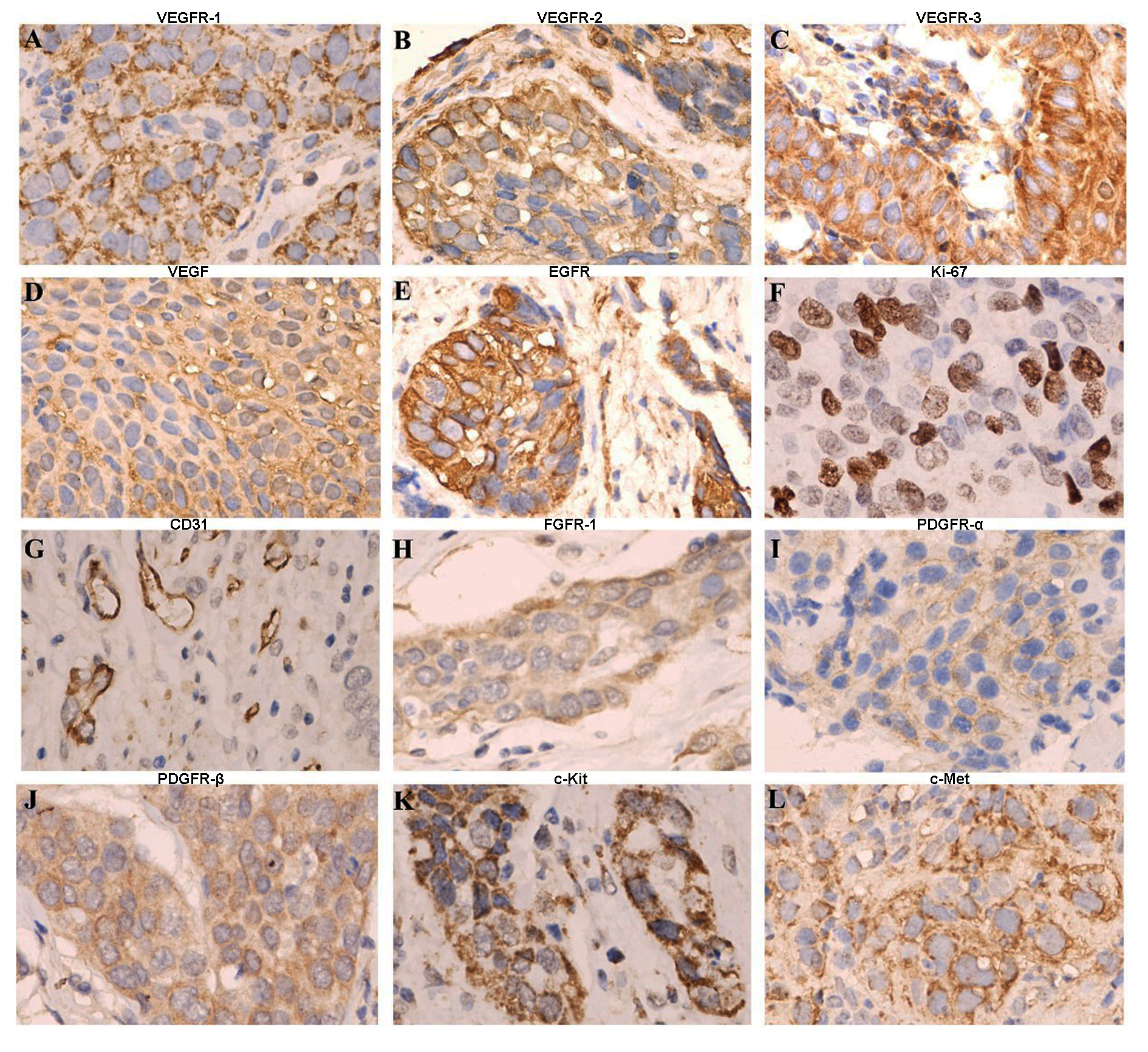

Supplement: Supplementary file 5 — Additional file 5: Figure S1. Representative immunohistochemical staining of 12 potential biomarkers [file 12916_2022_2649_MOESM5_ESM.tif]
