# Supplementary material for: A multi-center, single-arm, phase II study of anlotinib plus paclitaxel and cisplatin as the first-line therapy of recurrent/advanced esophageal squamous cell carcinoma
Source: BMC Med. 2022 Dec 8;20:472. doi: 10.1186/s12916-022-02649-x (PMC9733004; doi:10.1186/s12916-022-02649-x)
Supplement: Supplementary file 6 — Additional file 6. Supplementary Methods for immunohistochemical staining and scoring of potential biomarkers [file 12916_2022_2649_MOESM6_ESM.docx]

**Supplementary Methods. Immunohistochemical staining and scoring of potential biomarkers, including VEGF, VEGFR-1, VEGFR-2, VEGFR-3, EGFR, Ki67, CD31, FGFR-1, PDGFR-α, PDGFR-β, c-Kit, c-Met**

Paraffin sections underwent xylene dewaxing, gradient ethanol hydration, dewaxing, and PBS washing, then EDTA buffer (1:50, Shiji Kang wei, China) was used for antigen repairing and 3% H_2_O_2_ was used for reducing specific staining. After blocking (5% BSA in PBS) for 30 min at room temperature, samples were incubated with primary antibody (**Additional file 4** **Table S4**) at 4 ℃ overnight, and then with orseradish peroxidase (HRP)-IgG for 30 min at room temperature. Diaminobenzidine (DAB) staining was applied to visualize the target proteins and hematoxylin staining was used to re-stain the nucleus. The target protein was detected by 3,3'-Diaminobenzidine (DAB) staining, and the nucleus was re-stained using hematoxylin. The staining results were finally observed under a light microscope (Olympus, Japan). Two trained scientists independently performed blinded scoring. Once opinions diverged, the stained tissue sections were reevaluated until a consensus was reached.

The immunohistochemical staining score (H-score) was performed using semi-quantitative scoring and determined by the staining intensity and staining ratio [^1^](#_ENREF_1). The staining intensity was divided into 4 grades (grade 0-3): 0 = no staining; 1 = light yellow; 2 = brownish yellow; 3 = tan. The staining ratio was also divided into 4 grades (grade 0-3) according to the percentage of positive cells: 0 = no cell staining; 1 = if < 10% of stained cells; 2 = if ≤ 50% of stained cells; 3 = if > 50% of stained cells. The final H-score was obtained by multiplying the staining intensity with the staining ratio (H-score = staining intensity × staining ratio).

For CD31-assessed microvessel density (MVD), any brown-stained endothelial cells or clusters of endothelial cells that were separated from adjacent microvessels, tumor cells, and other connective tissue components were considered to be individual, countable microvessels. Vascular endothelial cells that may originate from the same vessel but were simply located differently are counted as separate vessels, whereas microvessels with lumens > 8 erythrocytes in diameter, with thicker myeloid or sclerotic areas, areas of sparse tumor cells, and areas close to normal tissue were not counted. At low magnification (100×), the tissue sections were observed, the area with the highest number of microvessels in the tumor was selected, the cancer tissue with the highest number of microvessels was stained (brown), and the number of microvessels in five fields of view was recorded at 200×, and the average was taken as the MVD value [^2^](#_ENREF_2)^,^ [^3^](#_ENREF_3). The median H-score and MVD values were selected as cut-off values [^4^](#_ENREF_4)^,^ [^5^](#_ENREF_5).

**Reference**

1. Dhakal HP, Naume B, Synnestvedt M, et al. Expression of vascular endothelial growth factor and vascular endothelial growth factor receptors 1 and 2 in invasive breast carcinoma: prognostic significance and relationship with markers for aggressiveness. *Histopathology* 2012;61:350-364.

2. Maeda K, Chung YS, Takatsuka S, et al. Tumor angiogenesis as a predictor of recurrence in gastric carcinoma. *Journal of clinical oncology : official journal of the American Society of Clinical Oncology* 1995;13:477-481.

3. Maeda K, Chung YS, Ogawa Y, et al. Prognostic value of vascular endothelial growth factor expression in gastric carcinoma. *Cancer* 1996;77:858-863.

4. Pajares MJ, Agorreta J, Larrayoz M, et al. Expression of tumor-derived vascular endothelial growth factor and its receptors is associated with outcome in early squamous cell carcinoma of the lung. *Journal of clinical oncology : official journal of the American Society of Clinical Oncology* 2012;30:1129-1136.

5. Decaussin M, Sartelet H, Robert C, et al. Expression of vascular endothelial growth factor (VEGF) and its two receptors (VEGF-R1-Flt1 and VEGF-R2-Flk1/KDR) in non-small cell lung carcinomas (NSCLCs): correlation with angiogenesis and survival. *The Journal of pathology* 1999;188:369-377.
