# Supplementary material for: A multi-center, single-arm, phase II study of anlotinib plus paclitaxel and cisplatin as the first-line therapy of recurrent/advanced esophageal squamous cell carcinoma
Source: BMC Med. 2022 Dec 8;20:472. doi: 10.1186/s12916-022-02649-x (PMC9733004; doi:10.1186/s12916-022-02649-x)
Supplement: Supplementary file 7 — Additional file 7: Table S5. Patients who achieved complete response (n = 4) [file 12916_2022_2649_MOESM7_ESM.docx]

**Table S5. Patients who achieved complete response (n=4)**

| Baseline characteristics | Case 1 | Case 2 | Case 3 | Case 4 |
| --- | --- | --- | --- | --- |
| Age (years) | 75 | 73 | 71 | 56 |
| Sex | Female | Male | Male | Female |
| ECOG PS | 0 | 1 | 1 | 0 |
| Clinical stage | IVb | IVb | IVb | IVb |
| Previous surgical treatment | Yes | Yes | Yes | Yes |
| Metastatic site | Yes | Yes | Yes | Yes |
| Distant Lymph node | Yes | Yes | Yes | Yes |
| Lung | Yes | No | No | No |
| Liver | No | No | Yes | No |
| Other metastatic sites | No | No | No | No |
| Number of metastatic sites | ≤ 2 | ≤ 2 | > 2 | ≤ 2 |
| Diameter of the target lesion (mm) | 11 | 17 | 50 | 16.79 |
| PFS (initial therapy, months) | 11.73 | 2.79^a^ | 8.08 | 6.93 |
| PFS (maintenance therapy, months) | 5.39 | NA | 3.42 | 2.33 |
| DoR (months) | 5.39 | 1.41 | 6.74 | 5.45 |
| Time to response (months) | 6.37 | 1.41 | 1.38 | 1.51 |

ECOG PS = Eastern Cooperative Oncology Group Performance Score; PFS = progression-free survival; DOR = duration of response.

^a^ represented loss to follow-up.
