# Supplementary material for: A multi-center, single-arm, phase II study of anlotinib plus paclitaxel and cisplatin as the first-line therapy of recurrent/advanced esophageal squamous cell carcinoma
Source: BMC Med. 2022 Dec 8;20:472. doi: 10.1186/s12916-022-02649-x (PMC9733004; doi:10.1186/s12916-022-02649-x)
Supplement: Supplementary file 8 — Additional file 8: Table S6. Subgroup analysis of the relation between clinical factors and progression-free survival (PFS) [file 12916_2022_2649_MOESM8_ESM.docx]

**Table S6.** **Subgroup analysis of the relation between clinical factors and progression-free survival (PFS)**

| Characteristics | No. of patients | Median PFS (months, 95% CI) | *p*-value |
| --- | --- | --- | --- |
| Age |  |  | 0.964 |
| ≤ 65 | 23 | 8.97 (4.45-13.49) |  |
| > 65 | 23 | 8.38 (7.48-9.29) |  |
| Sex |  |  | 0.756 |
| Male | 32 | 8.08 (6.41-9.75) |  |
| Female | 14 | 10.38 (6.44-14.32) |  |
| ECOG PS |  |  | 0.967 |
| 0 | 15 | 10.38 (5.79-14.97) |  |
| 1 | 31 | 8.08 (6.73-9.43) |  |
| Previous surgical treatment |  |  | 0.422 |
| Yes | 21 | 10.38 (6.76-14.00) |  |
| No | 25 | 7.59 (4.85-10.33) |  |
| Lymph node metastasis |  |  | 0.490 |
| Yes | 34 | 9.76 (7.14-12.38) |  |
| No | 12 | 6.37 (5.82-6.93) |  |
| Lung metastasis |  |  | 0.093 |
| Yes | 16 | 11.73 (7.28-16.18) |  |
| No | 30 | 7.59 (6.39-8.80) |  |
| Liver metastasis |  |  | 0.164 |
| Yes | 13 | 7.59 (4.94-10.24) |  |
| No | 33 | 8.97 (5.64-12.30) |  |
| Number of metastatic sites |  |  | 0.530 |
| ≤ 2 | 31 | 8.97 (5.45-12.49) |  |
| > 2 | 15 | 8.38 (7.29-9.47) |  |
| Distant metastasis |  |  | 0.347 |
| Yes | 41 | 8.97 (6.21-11.73) |  |
| No | 5 | 8.38 (4.32-12.45) |  |

ECOG PS = Eastern Cooperative Oncology Group Performance Score; CI = confidence interval.
