# Supplementary material for: A multi-center, single-arm, phase II study of anlotinib plus paclitaxel and cisplatin as the first-line therapy of recurrent/advanced esophageal squamous cell carcinoma
Source: BMC Med. 2022 Dec 8;20:472. doi: 10.1186/s12916-022-02649-x (PMC9733004; doi:10.1186/s12916-022-02649-x)
Supplement: Supplementary file 9 — Additional file 9: Table S7. Subgroup analysis of the relation between clinical factors and overall survival (OS) [file 12916_2022_2649_MOESM9_ESM.docx]

**Table S7. Subgroup analysis of the relation between clinical factors and overall survival (OS)**

| Characteristics | No. of patients | Median OS (95% CI) | *p*-value |
| --- | --- | --- | --- |
| Age |  |  | 0.587 |
| ≤ 65 | 23 | 18.53 (11.40-25.66) |  |
| > 65 | 23 | 15.08 (7.55-22.62) |  |
| Sex |  |  | 0.280 |
| Male | 32 | 16.43 (12.81-20.05) |  |
| Female | 14 | NA (NA-NA) |  |
| ECOG PS |  |  | 0.648 |
| 0 | 15 | 17.31 (7.18-27.44) |  |
| 1 | 31 | 18.53 (12.97-24.09) |  |
| Previous surgical treatment |  |  | 0.292 |
| Yes | 21 | 19.65 (11.32-27.98) |  |
| No | 25 | 17.31 (8.78-25.85) |  |
| Lymph node metastasis |  |  | 0.719 |
| Yes | 34 | 18.53 (8.95-28.11) |  |
| No | 12 | 19.65 (12.84-26.46) |  |
| Lung metastasis |  |  | 0.172 |
| Yes | 16 | 25.17 (NA-NA) |  |
| No | 30 | 16.43 (12.98-19.88) |  |
| Liver metastasis |  |  | 0.041 |
| Yes | 13 | 14.78 (6.39-23.17) |  |
| No | 33 | 23.03 (NA-NA) |  |
| Number of metastatic sites |  |  | 0.344 |
| ≤ 2 | 31 | 23.03 (13.17-32.89) |  |
| > 2 | 15 | 15.08 (8.61-21.55) |  |
| Distant metastasis |  |  | 0.957 |
| Yes | 41 | 18.53 (12.15-24.91) |  |
| No | 5 | 17.31 (0.33-34.29) |  |

ECOG PS = Eastern Cooperative Oncology Group Performance Score; CI = confidence interval.
