# Supplementary material for: A multi-center, single-arm, phase II study of anlotinib plus paclitaxel and cisplatin as the first-line therapy of recurrent/advanced esophageal squamous cell carcinoma
Source: BMC Med. 2022 Dec 8;20:472. doi: 10.1186/s12916-022-02649-x (PMC9733004; doi:10.1186/s12916-022-02649-x)
Supplement: Supplementary file 10 — Additional file 10: Table S8. Subgroup analysis of the correlation between clinical factors and overall response rate (ORR) [file 12916_2022_2649_MOESM10_ESM.docx]

**Table S8.** **Subgroup analysis of the correlation between clinical factors and overall response rate (ORR)**

| Characteristics | No. of patients | ORR (%, 95% CI) | *p*-value |
| --- | --- | --- | --- |
| Age |  |  |  |
| ≤ 65 | 23 | 19 (82.6%, 61.2%-95.0%) | 0.300 |
| > 65 | 23 | 16 (69.6%, 47.1%-86.8%) |  |
| Sex |  |  | 0.524 |
| Male | 32 | 23 (71.9%, 53.3%-86.3%) |  |
| Female | 14 | 12 (85.7%, 57.2%-98.2%) |  |
| ECOG PS |  |  | 0.949 |
| 0 | 15 | 12 (80.0%, 51.9%-95.7%) |  |
| 1 | 31 | 23 (74.2%, 55.4%-88.1%) |  |
| Previous surgery |  |  | 0.161 |
| Yes | 21 | 18 (85.7%, 63.7%-97.0%) |  |
| No | 25 | 17 (68.0%, 46.5%-85.1%) |  |
| Lymph node metastasis |  |  | 0.620 |
| Yes | 34 | 27 (79.4%, 62.1%-91.3%) |  |
| No | 12 | 8 (66.7%, 34.9%-90.1%) |  |
| Lung metastasis |  |  | > 0.999 |
| Yes | 16 | 12 (75.0%, 47.6%-92.7%) |  |
| No | 30 | 23 (76.7%, 57.7%-90.1%) |  |
| Liver metastasis |  |  | 0.764 |
| Yes | 13 | 9 (69.2%, 38.6%-90.9%) |  |
| No | 33 | 26 (78.8%, 61.1%-91.0%) |  |
| Number of metastatic sites |  |  | 0.124 |
| ≤ 2 | 31 | 21 (67.7%, 48.6%-83.3%) |  |
| > 2 | 15 | 14 (93.3%, 68.1%-99.8%) |  |
| Distant metastasis |  |  | > 0.999 |
| Yes | 41 | 31 (75.6%, 59.7%-87.6%) |  |
| No | 5 | 4 (80.0%, 28.4%-99.5%) |  |

ECOG PS = Eastern Cooperative Oncology Group Performance Score; CI = confidence interval.
