# Supplementary material for: A multi-center, single-arm, phase II study of anlotinib plus paclitaxel and cisplatin as the first-line therapy of recurrent/advanced esophageal squamous cell carcinoma
Source: BMC Med. 2022 Dec 8;20:472. doi: 10.1186/s12916-022-02649-x (PMC9733004; doi:10.1186/s12916-022-02649-x)
Supplement: Supplementary file 11 — Additional file 11: Table S9. Multivariate analysis of the correlation between biomarkers and progression-free survival (PFS) [file 12916_2022_2649_MOESM11_ESM.docx]

**Table S9. Multivariate analysis of the correlation between biomarkers and progression-free survival (PFS)**

| Characteristics | No. of patients | Univariate analysis | | | Multivariate analysis | | |
| --- | --- | --- | --- | --- | --- | --- | --- |
|  |  | HR | 95% CI | *p*-value | HR | 95% CI | *p*-value |
| VEGFR-1 (≤ 5 vs. > 5) | 11 vs. 11 | 0.404 | 0.137-1.185 | 0.099 | 0.306 | 0.074-1.257 | 0.100 |
| VEGFR-2 (≤ 2 vs. > 2) | 15 vs. 7 | 0.744 | 0.200-2.771 | 0.659 |  |  |  |
| VEGFR-3 (≤ 6 vs. > 6) | 16 vs. 6 | 0.968 | 0.328-2.855 | 0.953 |  |  |  |
| VEGF (≤ 2 vs. > 2) | 14 vs. 6 | 0.270 | 0.058-1.252 | 0.094 | 0.226 | 0.025-2.037 | 0.185 |
| EGFR (≤ 2 vs. > 2) | 13 vs. 9 | 0.823 | 0.291-2.327 | 0.713 |  |  |  |
| Ki67 (≤ 20% vs. > 20%) | 12 vs. 11 | 0.946 | 0.345-2.595 | 0.914 |  |  |  |
| CD31 (≤ 6 vs. > 6) | 12 vs. 11 | 0.355 | 0.107-1.181 | 0.091 | 0.768 | 0.204-2.900 | 0.697 |
| FGFR-1 (≤ 2 vs. > 2) | 15 vs. 8 | 0.732 | 0.262-2.045 | 0.552 |  |  |  |
| PDGFR-α (≤ 1 vs. > 1) | 14 vs. 7 | 0.489 | 0.144-1.662 | 0.252 |  |  |  |
| PDGFR-β (≤ 2 vs. > 2) | 18 vs. 3 | 1.134 | 0.243-5.291 | 0.873 |  |  |  |
| c-Kit (≤ 4 vs. > 4) | 17 vs. 6 | 0.172 | 0.036-0.829 | 0.028 | 0.032 | 0.002-0.606 | 0.022 |
| c-Met (≤ 4 vs. > 4) | 23 vs. 0 | -- | -- | -- |  |  |  |

VEGFR = vascular endothelial growth factor receptor; FGFR = fibroblast growth factor receptor; PDGFR = platelet-derived growth factor receptor; VEGR = vascular endothelial growth factor; EGFR = epidermal growth factor receptor; HR = hazard ratio; CI = confidence interval.

The cutoff values for low versus high biomarker expressions were the median H score or staining percentage (Ki67) or microvessel density (MVD, CD31) for the study population evaluable for biomarker.
