# Supplementary material for: A multi-center, single-arm, phase II study of anlotinib plus paclitaxel and cisplatin as the first-line therapy of recurrent/advanced esophageal squamous cell carcinoma
Source: BMC Med. 2022 Dec 8;20:472. doi: 10.1186/s12916-022-02649-x (PMC9733004; doi:10.1186/s12916-022-02649-x)
Supplement: Supplementary file 12 — Additional file 12: Table S10. Multivariate analysis of the correlation between biomarkers and overall survival (OS) [file 12916_2022_2649_MOESM12_ESM.docx]

**Table S10. Multivariate analysis of the correlation between biomarkers and overall survival (OS)**

| Characteristics | No. of patients | Univariate analysis | | | | Multivariate analysis | | |
| --- | --- | --- | --- | --- | --- | --- | --- | --- |
|  |  | HR | 95% CI | | *p*-value | HR | 95% CI | *p*-value |
| VEGFR-1 (≤ 5 vs. > 5) | 11 vs. 11 | 0.441 | | 0.128-1.514 | 0.193 |  |  |  |
| VEGFR-2 (≤ 2 vs. > 2) | 15 vs. 7 | 0.204 | | 0.026-1.600 | 0.130 |  |  |  |
| VEGFR-3 (≤ 6 vs. > 6) | 16 vs. 6 | 0.496 | | 0.106-2.310 | 0.371 |  |  |  |
| VEGF (≤ 2 vs. > 2) | 14 vs. 6 | 0.023 | | 0.000-4.110 | 0.154 |  |  |  |
| EGFR (≤ 2 vs. > 2) | 13 vs. 9 | 0.268 | | 0.058-1.246 | 0.093 | 0.375 | 0.074-1.892 | 0.235 |
| Ki67 (≤ 20% vs. > 20%) | 12 vs. 11 | 1.255 | | 0.391-4.031 | 0.703 |  |  |  |
| CD31 (≤ 6 vs. > 6) | 12 vs. 11 | 0.396 | | 0.112-1.405 | 0.152 |  |  |  |
| FGFR-1 (≤ 2 vs. > 2) | 15 vs. 8 | 0.266 | | 0.058-1.224 | 0.089 | 0.398 | 0.079-2.014 | 0.266 |
| PDGFR-α (≤ 1 vs. > 1) | 14 vs. 7 | 0.535 | | 0.139-2.057 | 0.363 |  |  |  |
| PDGFR-β (≤ 2 vs. > 2) | 18 vs. 3 | 2.351 | | 0.455-12.151 | 0.308 |  |  |  |
| c-Kit (≤ 4 vs. > 4) | 17 vs. 6 | 0.279 | | 0.056-1.376 | 0.117 |  |  |  |
| c-Met (≤ 4 vs. > 4) | 23 vs. 0 | -- | | -- | -- |  |  |  |

VEGFR = vascular endothelial growth factor receptor; FGFR = fibroblast growth factor receptor; PDGFR = platelet-derived growth factor receptor; VEGR = vascular endothelial growth factor; EGFR = epidermal growth factor receptor; HR = hazard ratio; CI = confidence interval.

The cutoff values for low versus high biomarker expressions were the median H score or staining percentage (Ki67) or microvessel density (MVD, CD31) for the study population evaluable for biomarker.
